# CXCL13 in idiopathic pulmonary arterial hypertension and chronic thromboembolic pulmonary hypertension

**DOI:** 10.1186/s12931-016-0336-5

**Published:** 2016-02-29

**Authors:** Karen M. Olsson, Sandra Olle, Jan Fuge, Tobias Welte, Marius M. Hoeper, Christian Lerch, Lavinia Maegel, Hermann Haller, Danny Jonigk, Lena Schiffer

**Affiliations:** Department of Respiratory Medicine and German Center of Lung Research (DZL), Hannover Medical School, Hannover, Germany; Department of Pediatric Nephrology, Hannover Medical School, Hannover, Germany; Department of Pathology, Hannover Medical School, Hannover, Germany; Department of Nephrology, Hannover Medical School, Hannover, Germany

**Keywords:** Hypertension, Pulmonary, CXCL13, Chronic thromboembolic pulmonary hypertension, Biomarker, Inflammation

## Abstract

**Background:**

Chemokine CXC ligand 13 (CXCL13) has been implicated in perivascular inflammation and pulmonary vascular remodeling in patients with idiopathic pulmonary artery hypertension (IPAH). We wondered whether CXCL13 may also play a role in chronic thromboembolic pulmonary hypertension (CTEPH) and whether serum levels of CXCL13 might serve as biomarkers in these conditions.

**Methods:**

Lung tissue from patients with IPAH or CTEPH was immunostained for CXCL13. Serum samples were obtained from patients with IPAH (*n* = 42) or CTEPH (*n* = 50) and from healthy controls (*n* = 13). Serum CXCL13 concentrations were measured by enzyme-linked immunosorbent assay technology and were evaluated for associations with markers of disease severity and survival.

**Results:**

CXCL13 was expressed in pulmonary vascular lesions and lymphocytes of patients with IPAH and inoperable CTEPH, respectively. Serum CXCL13 was elevated in patients compared to healthy controls [median, interquartile range, 83 (55,114) pg/ml versus 40 (28, 48) pg/ml; *p* < 0.001]. Serum CXCL13 showed only weak and inconsistent correlations with markers of inflammation or disease severity. In both populations, patients with serum CXCL13 above the median of the respective groups did not have a higher risk of death than patients with lower serum CXCL13.

**Conclusions:**

CXCL13 was overexpressed in pulmonary vascular lesions of patients with IPAH and CTEPH, and increased serum concentrations were found in patients with IPAH and CTEPH, suggesting a potential pathogenic role of CXCL13 in both diseases. However, given the weak associations between serum CXCL13 and markers of disease severity and outcome, CXCL13 is unlikely to become a promising biomarker in these patient populations.

## Background

Idiopathic pulmonary arterial hypertension (PAH) and chronic thromboembolic pulmonary hypertension (CTEPH) are diseases characterized by a progressive increase in pulmonary vascular resistance due to an obliterative pulmonary vasculopathy which eventually results in right heart failure and death, if not effectively treated [1, 2].

The pathogenesis of pulmonary vascular remodeling in both IPAH and CTEPH is not completely understood, but an inflammatory component has been noted in both conditions [3–7]. Perivascular accumulation of B-type and T-type lymphocytes has been demonstrated in the lungs of patients with PAH as well as CTEPH [6, 8], and even pulmonary lymphoid neogenesis was demonstrated in patients with IPAH [9].

Chemokine CXC ligand 13 (CXCL13), also known as C-X-C motif chemokine 13, B-lymphocyte-chemoattractant (BLC) or B-cell-attracting chemokine-1 (BAC-1) is a CXC subtype member of the chemokine superfamily [10]. The receptor of CXCL13, C-X-C chemokine receptor type 5 (CXCR5), is expressed on mature B-cells and follicular T-helper cells [10]. CXCL13 and CXCR5 are key players in the trafficking of B-cells, and CXCL13 serum levels are increased in various autoimmune disorders as well as in patients with idiopathic pulmonary fibrosis [11]. Perros et al. have recently shown that CXCL13 mRNA is strongly expressed and co-located with lymphoid neogenesis in lung tissue from patients with IPAH [9].

The objectives of the present study were (i) to confirm the findings by Perros et al. [9] in patients with IPAH, (ii) to demonstrate whether increased CXCL13 activity can also be found in lungs from patients with CTEPH and (iii) to investigate whether serum levels of CXCL13 might be used as biomarkers of disease severity in these patient populations.

## Methods

### Patients and controls

Patients diagnosed with IPAH or inoperable CTEPH between January 1st, 2007 and December 31st, 2014 were eligible for this study. Follow-up ended April 15th, 2015. The diagnoses of both conditions were made according to standard criteria [12] and included ventilation/perfusion scintigraphy and right heart catheterization in all patients. For the assessment of CTEPH, pulmonary angiography was mandatory. A multidisciplinary team of experts made the decision about operability. Patients with post-capillary pulmonary hypertension, connective tissue disease and significant parenchymal lung disease were excluded from this study.

Blood samples from healthy volunteers were collected as controls. Written informed consent was provided from all patients and controls and the Hannover Medical School ethics committee (Ethikkommission der Medizinischen Hochschule Hannover) approved this study.

### Right heart catheterization

Right heart catheterizations were performed via a jugular approach following a standardized protocol. The pressure transducer was zeroed at the mid-thoracic level. Measurements included right atrial pressure, mean pulmonary arterial pressure (PAPm), pulmonary arterial wedge pressure (PAWP) and mixed venous oxygen saturation (SvO_2_). Cardiac output (CO) was measured by thermodilution with the reported value being the average of at least three recordings with less than 10 % variation. PVR was calculated according to standard formula.

### Measurements of circulating CXCL13 levels

Venous blood for quantification of circulating CXCL13 levels was obtained from patients with IPAH and CTEPH at the time of right heart catheterization. The blood samples were immediately placed on ice, centrifuged at 1000 *g* for 10 min and stored at −80 °C. Serum concentrations of CXCL13 were quantified by the commercially available Quantikine enzyme-linked immunosorbent essay (ELISA) methodology according to the manufacturer’s instructions (R&D Systems, Minneapolis, MN, USA, Catalog Number DCX130). All samples were measured in duplicates. Color intensity was measured by a standard ELISA Reader (Tecan spectra mini, Crailsheim, Germany). Quantikine kit standards were used for construction of standard curves.

### Immunohistochemical staining

Explanted lungs were available from three patients with IPAH and one patient with CTEPH. Lung tissue from three organ donors was used as control. Serially cut slides of formalin-fixed and paraffin embedded lung tissues were immunohistochemically stained for CXCL13 using polyclonal antibody (LS-C104337; LSBio, Seattle WA, USA) following our established standard ABC protocol [13].

### Statistical analysis

Data are shown as mean ± standard deviation (SD). For comparison of the study populations, Fisher’s exact test, Chi-square test and two-sided paired *t*-test were used as appropriate. Potential associations of CXCL13 with clinical and hemodynamic variables were assessed with Pearson’s correlation analysis and two-sided testing for significance. Kaplan-Meier estimates on survival were made for patients with IPAH and CTEPH based on their CXCL13 levels above or below the median of the respective groups. Comparisons were made by log-rank analysis. *P*-values <0.05 were considered statistically significant.

## Results

The study enrolled 92 patients (IPAH, n = 42; CTEPH, *n* = 50) and 13 healthy controls. The demographics and baseline characteristics are shown in Table 1.

**Table 1:** Characteristics of patients and controls at inclusion

	All (IPAH + CTEPH) (n = 92)	IPAH (n = 42)	CTEPH (n = 50)	Healthy controls (n = 13)	*P*-value^*^	*P*-value^**^
Age (years)	59 ± 17	49 ± 17	68 ± 12	46 ± 16	<0.01	0.010
Female (%)	54	67	44	54	0.030	0.973
Body mass index (kg/m^2^)	27 ± 6	28 ± 7	26 ± 5	23 ± 3	0.259	0.009
NYHA II/III/IV (n)	0/10/76/6	0/7/33/2	0/3/43/4	n.a.	n.a.	n.a.
6 min walking distance (m)	330 ± 122	350 ± 115	314 ± 126	n.a	0.165	n.a.
Right atrial pressure (mmHg)	7 ± 5	8 ± 5	5 ± 4	n.a	0.004	n.a.
Mean pulmonary artery pressure (mmHg)	47 ± 14	53 ± 14	41 ± 10	n.a	<0.01	n.a.
Pulmonary arterial wedge pressure (mmHg)	8 ± 3	8 ± 3	8 ± 3	10	0.638	n.a.
Cardiac output (L/min)	4.3 ± 1.2	4.3 ± 1.4	4.4 ± 1.2	n.a	0.733	n.a.
Cardiac index (L/min/m^2^)	2.3 ± 0.6	2.3 ± 0.7	2.3 ± 0.5	n.a	0.955	n.a.
Pulmonary vascular resistance (dyn · s · cm^−5^)	802 ± 416	970 ± 455	661 ± 321	n.a	<0.01	n.a.
Mixed venous oxygen saturation (%)	63 ± 7	63 ± 8	63 ± 6	n.a	0.991	n.a.
CXCL13 (pg/ml)	120 ± 124	122 ± 127	118 ± 124	42 ± 15	0.858	0.026
CXCL13 (pg/ml) Median (IQR)	83 (55,114)	81 (64,113)	84 (51,120)	40 (28,48)	0.597^***^	<0.001^***^
CRP (mg/l)	9 ± 15	8 ± 10	10 ± 17	n.a	0.583	n.a.
S-Sodium (mmol/l)	140 ± 3	140 ± 3	140 ± 3	n.a	0.259	n.a.
NT-proBNP (ng/l)	2,530 ± 2,222	2,761 ± 2,165	2,333 ± 2,278	n.a	0.412	n.a.
NT-proBNP (ng/l) Median, IQR	1,929 (802–3,413)	1,995 (1,276-4,010)	1,747 (658–3,257)	n.a	0.222^***^	n.a.
Bilirubin (μmol/l)	15 ± 9	17 ± 9	14 ± 9	n.a	0.069	n.a.
Uric acid (μmol/l)	411 ± 154	415 ± 142	407 ± 167	n.a	0.876	n.a.
Creatinine (μmol/l)	90 ± 24	85 ± 26	94 ± 22	n.a	0.076	n.a.
eGFR ml/min/1,73 m^2^	75 ± 26	83 ± 31	69 ± 18	n.a	0.008	n.a.

^*^IPAH vs. CTEPH, ^**^IPAH and CTEPH combined versus controls

*n.a*. not available, ^***^Mann-Whitney *U*-test

### CXCL13 serum concentrations in patients with IPAH and CTEPH

**Fig. 1:**
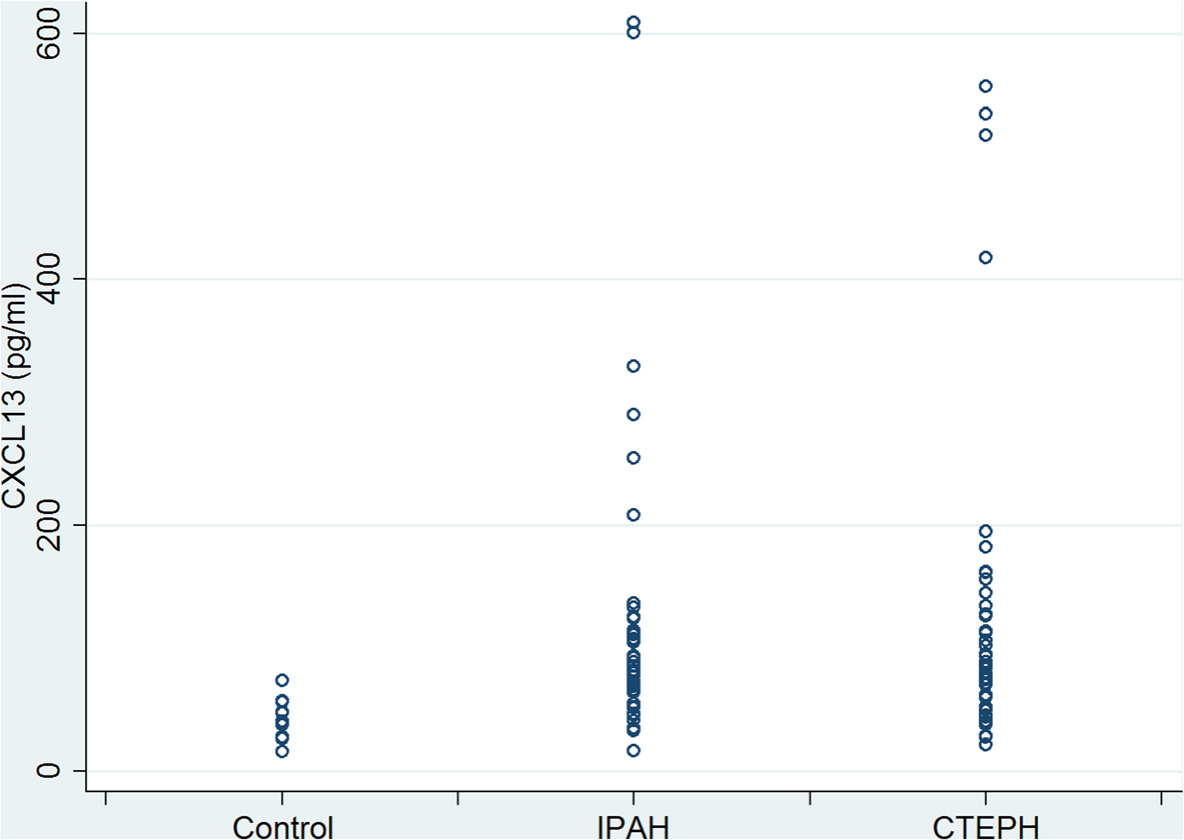
Box plot showing serum CXCL13 in healthy controls compared to patients with idiopathic pulmonary arterial hypertension (IPAH) and patients with chronic thromboembolic pulmonary hypertension (CTEPH)

As shown in Table 1 and Fig. 1, serum CXCL13 was elevated in the patient cohort compared to the control group (120 ± 124 pg/ml versus 42 ± 15 pg/ml; *p* = 0.026). There were no significant differences in CXCL13 levels between IPAH and CTEPH patients (122 ± 127 pg/ml versus 118 ± 124 pg/ml; *p* = 0.858).

### Correlations between serum CXCL13 and markers of disease severity

In the whole group (IPAH and CTEPH combined), there were no significant correlations of serum CXCL13 with clinical, laboratory and hemodynamic parameters (data not shown). As shown in Table 2, patients with IPAH showed a significant correlation between CXCL13 and CRP; which was, however, not found in patients with CTEPH. In the latter group, there were statistically significant correlations between CXCL13 and right atrial pressure, cardiac output, cardiac index and PVR, respectively (Table 3). These correlations were relatively weak and were not seen in patients with IPAH. Patients with particular high serum CXCL13 (>200 pg/ml; Fig. 1) did not differ from the rest of the cohort in terms of age, sex or hemodynamics (data not shown).

**Table 2:** Correlations of serum CXCL13 with clinical, hemodynamic and laboratory variables in patients with idiopathic pulmonary arterial hypertension

	r	*p*
CRP	0.470	0.003

**Table 3:** Correlations of serum CXCL13 with clinical, hemodynamic and laboratory variables in patients with chronic thromboembolic pulmonary hypertension

	r	*p*
RAP	0.317	0.025
CO	−0.286	0.044
CI	−0.279	0.049
PVR	0.314	0.026

### Serum CXCL13 and survival

**Fig. 2:**
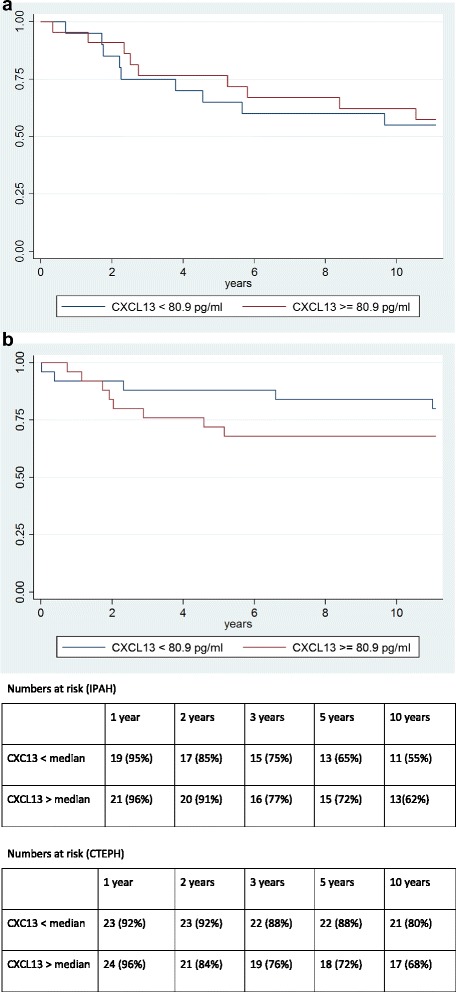
**a** Kaplan Meier Analysis showing probability of survival in patients with idiopathic pulmonary arterial hypertension according to serum CXCL13 above or below the median group value. The difference between both groups was not statistically significant (*p* = 0.786 by Log-Rank testing). **b** Kaplan Meier Analysis showing probability of survival in patients with chronic thromboembolic pulmonary hypertension according to serum CXCL13 above or below the median group value. The difference between both groups was not statistically significant (*p* = 0.330 by Log-Rank testing)

As shown in Fig. 2a and b, patients with higher serum CXCL13 as defined by levels above the respective group medians did not have a higher mortality risk compared to their counterparts with lower serum CXCL13 (*p* = 0.786 for the IPAH cohort and *p* = 0.330 for the CTEPH cohort).

### CXCL13 expression in lungs from patients with IPAH and CTEPH

**Fig. 3:**
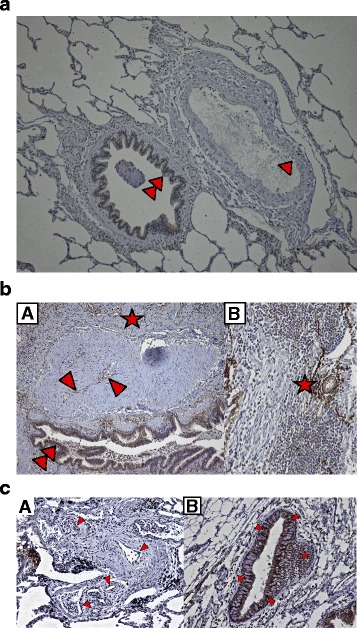
**a**. Immunostaining for CXCL13 in an explanted lung from an organ donor showing weak positivity in bronchial epithelium (double arrow heads *a*), but not in pulmonary vascular endothelial cells (arrow heads *a*), [Original magnification, A × 100, B × 200]. **b**. Immunostaining for CXCL13 in an explanted lung from a patient with idiopathic pulmonary hypertension showing positivity in pulmonary vascular endothelial cells (arrow heads A), bronchial epithelium (double arrow heads *a*) and perivascular lymphocytes (*a, *b) [Original magnification, A × 100, B × 200]. **c**. Immunostaining for CXCL13 in an explanted lung from a patient with chronic thromboembolic pulmonary hypertension showing positivity in pulmonary vascular endothelial cells (arrow heads *a*), bronchial epithelium (arrow heads *b*) and peribronchial lymphocytes (*b) [Original magnification, × 200]

Immunohistochemical CXCL13 staining of the explanted lungs from the controls showed weak positivity in bronchial epithelium but not in pulmonary vascular endothelial cells. Perivascular lymphocytes were not visible (Fig. 3a).

Immunohistochemical CXCL13 staining of explanted lungs from patients with IPAH (Fig. 3b) and CTEPH (Fig. 3c) showed positivity in pulmonary vascular endothelial cells, bronchial epithelium, lymphatic vessels and perivascular lymphocytes.

## Discussion

Our findings confirm a previous study from Perros et al. showing that CXCL13 is expressed in the pulmonary vasculature of patients with IPAH. We extend these findings by showing that CXCL13 was also expressed in the pulmonary vasculature of a patient with CTEPH. In addition, we found increased serum CXCL13 in both patient populations compared to healthy controls. However, serum CXCL13 did show only weak and inconsistent correlations with markers of inflammation and disease severity in patients with IPAH and CTEPH. Furthermore, elevated serum CXCL13, i.e. CXCL13 concentrations above the median of the respective group, were not associated with worse survival. This is in contrast to the findings of Vuga et al. in patients with idiopathic pulmonary fibrosis (IPF) [11].

Our findings support the notion that inflammation is involved in the pathogenesis of IPAH and CTEPH. More specifically, they support a previous study by Perros et al. [9], which suggested that the lymphocytic inflammation of pulmonary vessels seen in IPAH is at least partly mediated by CXCL13. In addition, our data indicate that similar mechanisms may play a role in CTEPH. The role of CXCL13 and its receptor CXCR5 for lymphocyte migration was described in gene targeted mice more than a decade ago [14, 15], and was confirmed in different human diseases with inflammatory components [11, 16, 17].

Although not formally proven, it is likely that the elevated serum levels of CXCL13 found in patients with IPAH and CTEPH result from a spillover of CXCL13 overexpressed in the lungs. We did not observe relevant associations between serum CXCL13 and markers of disease severity or mortality in both patient populations. This does not exclude the possibility that serum CXCL13 might reflect disease activity, but our study design did not allow us to explore this hypothesis.

Our study has several limitations including the retrospective design and the single center setting. Lung tissue was available from only a few patients. We do not consider this a major problem in IPAH as our data were merely meant to confirm a previous study [9]. However, lung tissue was available only from a single patient with CTEPH as lung transplantation is performed rarely in these patients. This finding awaits further confirmation by others. In addition, we had no information on the BMPR2 mutation status in our patient cohorts. The sample sizes for serum CXCL13 analyses in patients with IPAH and CTEPH were relatively small. Larger series might have yielded statistically significant associations with serum CXCL13 and markers of disease severity and survival. However, it is unlikely that larger studies would have resulted in a substantial change of our main findings.

## Conclusions

Our findings support the notion that CXCL13 might be associated with pulmonary vascular remodeling in IPAH and CTEPH, but it is unlikely that serum CXCL13 will be a useful biomarker in these conditions.

## Abbreviations

CI: cardiac index
CO: cardiac output
CRP: C-reactive protein
CTEPH: chronic thromboembolic pulmonary hypertension
CXCL13: chemokine CXC ligand 13
IPAH: idiopathic pulmonary arterial hypertension
PAH: pulmonary arterial hypertension
PAPm: mean pulmonary artery pressure
PAWP: pulmonary arterial wedge pressure
PH: pulmonary hypertension
PVR: pulmonary vascular resistance
RAP: right atrial pressure
